# Reverse transduction measured in the living cochlea by low-coherence heterodyne interferometry

**DOI:** 10.1038/ncomms10282

**Published:** 2016-01-06

**Authors:** Tianying Ren, Wenxuan He, Peter G. Barr-Gillespie

**Affiliations:** 1 Oregon Hearing Research Center, Department of Otolaryngology, Oregon Health & Science University, Portland, Oregon 97239, USA

## Abstract

It is generally believed that the remarkable sensitivity and frequency selectivity of mammalian hearing depend on outer hair cell-generated force, which amplifies sound-induced vibrations inside the cochlea. This ‘reverse transduction' force production has never been demonstrated experimentally, however, in the living ear. Here by directly measuring microstructure vibrations inside the cochlear partition using a custom-built interferometer, we demonstrate that electrical stimulation can evoke both fast broadband and slow sharply tuned responses of the reticular lamina, but only a slow tuned response of the basilar membrane. Our results indicate that outer hair cells can generate sufficient force to drive the reticular lamina over all audible frequencies in living cochleae. Contrary to expectations, the cellular force causes a travelling wave rather than an immediate local vibration of the basilar membrane; this travelling wave vibrates in phase with the reticular lamina at the best frequency, and results in maximal vibration at the apical ends of outer hair cells.

## Abstract (web summary)

Mammalian hearing is remarkable for its sensitivity and frequency selectivity. Here, the authors show that outer hair cell-generated force, which amplifies sound-induced vibrations inside the cochlea, is responsible for these traits.

## Graphical abstract

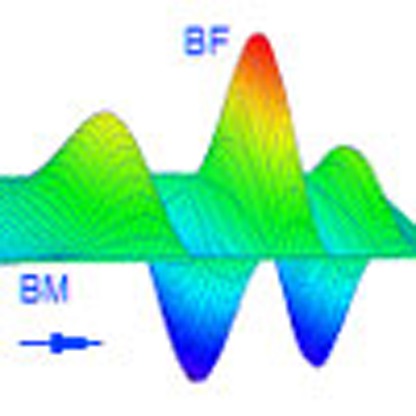

The electromechanical feedback system of the mammalian cochlea amplifies sound-induced vibrations using force generated by outer hair cells^1^,^2^,^3^,^4^,^5^,^6^,^7^,^8^,^9^,^10^,^11^ through somatic and hair-bundle motility^12^,^13^,^14^,^15^,^16^,^17^,^18^,^19^,^20^; such force is required for the extreme sensitivity and precise frequency selectivity of hearing. ‘Reverse transduction' force production has been shown *in vitro* by recording responses of the organ of Corti to electrical stimulation^21^,^22^. Because the cochlear feedback is not functional in isolated cochleae^23^,^24^,^25^,^26^,^27^,^28^, whether reverse transduction operates in living cochleae remains speculative. *In vivo* recordings of cochlear mechanical responses to electrical stimulation^29^,^30^,^31^,^32^ have previously only measured the movement of the basilar membrane, distant from outer hair cells. To investigate reverse transduction *in vivo*, we electrically stimulated the outer hair cells by passing current through the cochlear partition and measured vibrations of the reticular lamina, which is at the apical surfaces of the outer hair cells (Fig. 1a). Using a custom-built scanning heterodyne low-coherence interferometer (Methods), we demonstrate outer hair cell-driven reticular lamina vibration over the entire range of hearing frequencies in living mouse cochleae.

## Results

### Electrically evoked vibrations in wild-type mice

**Figure 1:**
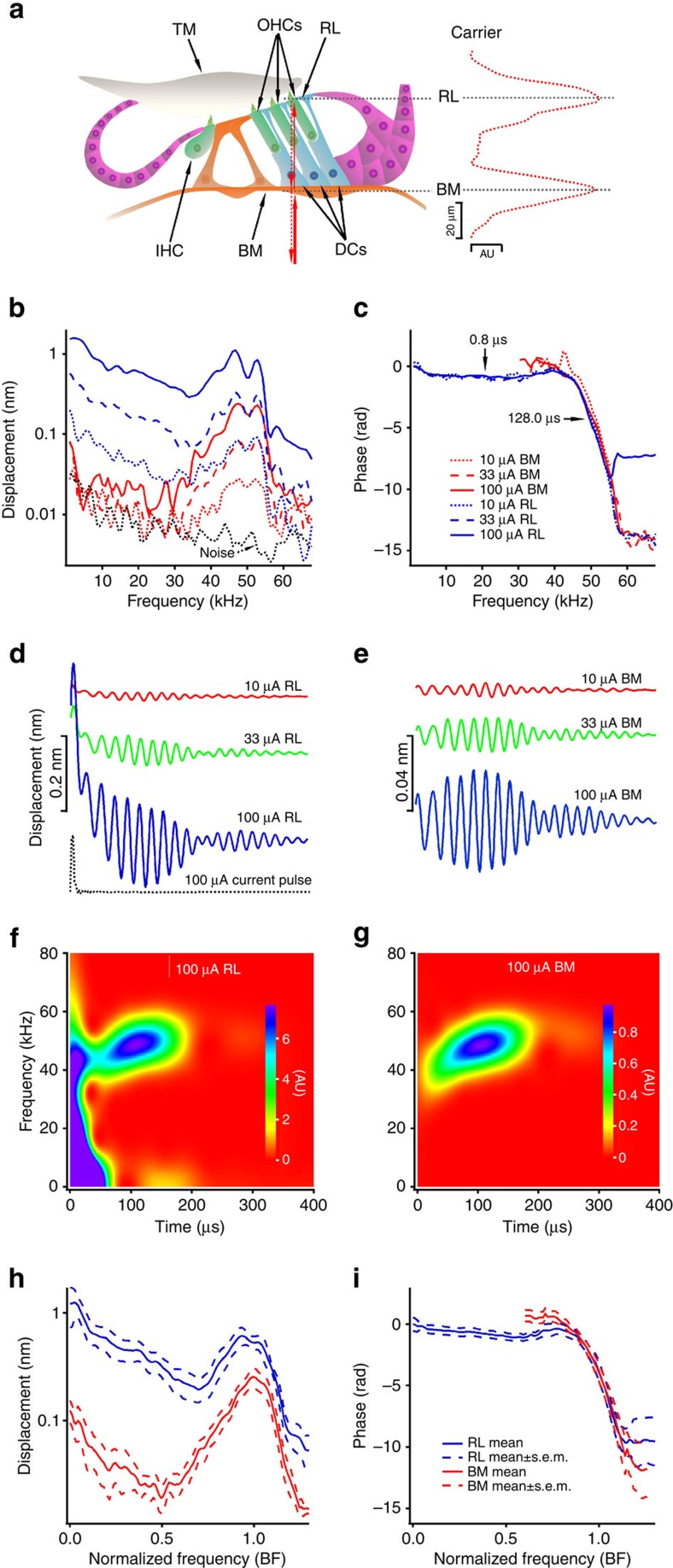
Measurement diagram and electrically evoked reticular lamina and basilar membrane vibrations in wild-type mice. (**a**) Diagram of the organ of Corti and measurement of the reticular lamina (RL) and basilar membrane (BM) vibrations. The BM and RL locations in the transverse direction were indicated by the peaks of the carrier signal (red dotted line on the right). IHC, inner hair cell. OHCs, outer hair cells. TM, tectorial membrane. DCs, Deiters' cells. (**b**) Magnitudes of RL (blue) and BM (red) responses as a function of frequency. The noise floor (black dotted line) was below 0.01 nm at frequencies above 15 kHz. (**c**) RL and BM phase. (**d**) Time responses of the RL (blue, green, and red curves) and the electrical stimulus (black dotted line). (**e**) Time responses of the BM. (**f**,**g**) Spectrograms of RL and BM responses to 100-μA current. (**h**,**i**) Grouped magnitudes and phase of RL (blue) and BM (red) responses to 100-μA currents (*n*=5; mean±s.e.m.). Frequency axes were normalized to the best frequency (BF) of the basilar membrane for calculating means and s.e.m.

When we applied a sinusoidal current stimulus of 100 μA, the reticular lamina vibrated with a magnitude of >1 nm at frequencies below 5 kHz (Fig. 1b, solid blue line), and substantial vibrations were detected up to 67.5 kHz. The magnitude response had a complex pattern with a clear peak at ∼48 kHz (that is, the best frequency, BF), and decreased proportionally with the stimulus level by ∼10 dB per step at all frequencies, as indicated by blue dashed and dotted lines. The noise level (black dotted line) was below 0.01 nm at frequencies above 15 kHz. The phase response of the reticular lamina (blue lines in Fig. 1c) showed two distinct parts: a flat region at frequencies below 40 kHz and above 55 kHz; and a steep region near the BF. Drastically different phase slopes indicate that the delay of reticular lamina vibration near the peak frequency (∼128 μs) is much larger than those at other frequencies (∼0.8 μs).

In contrast to the reticular lamina response, the basilar membrane vibration was small except at the BF (red lines in Fig. 1b). No broadband response was detected. At frequencies below 35 kHz, the basilar membrane vibration stimulated by 100-μA current was <1/20th of the reticular lamina vibration. Above 35 kHz, the magnitude increased to a peak at approximately the same frequency as the reticular lamina vibration, then decreased at higher frequencies. The peak magnitude was significantly smaller than that of the reticular lamina, and decreased linearly with the current level, while the asymmetric shape of the peak remained. Surprisingly, the phase curves of the basilar membrane vibration (red lines in Fig. 1c) overlapped with those of the reticular lamina vibration (blue lines) at the BF, indicating that the two structures moved synchronously in the same direction.

Time waveforms derived from Fig. 1b,c (see Methods) showed that, on the onset of the electrical impulse at time zero (Fig. 1d, black dotted line), the reticular lamina moved with negligible delay towards the scala tympani, then quickly returned to its equilibrium position when the impulse stopped, forming an initial large peak (Fig. 1d, blue line). Before returning to the resting position, a periodic oscillation started. This delayed response reached its maximum at ∼100 μs and gradually decreased to a minimum at ∼200 μs. A second episode of small periodic oscillations occurred between 200 and 350 μs. The magnitude of the time waveforms decreased linearly with current level (green and red lines in Fig. 1d,e). The exponential decay with a time constant of ∼28 μs (solid blue line in Fig. 1d) likely resulted from a decrease in the outer hair cell's membrane potential. Except for the lack of initial peak, the delayed periodic vibrations of the basilar membrane (Fig. 1e) were similar to those of the reticular lamina.

A spectrogram showed that the reticular lamina fast response occurred immediately on impulse onset and extended to >60 kHz, while the delayed response was detected only near the BF (Fig. 1f). In addition, the instantaneous frequency of the delayed response increased with time^33^,^34^,^35^,^36^,^37^ and reached the BF at ∼100 μs. By contrast, the spectrogram of the basilar membrane vibration showed only the delayed component near the BF (Fig. 1g). Frequency responses, time waveforms, and spectrograms were similar across animals. Grouped data from 100-μA stimulation (Fig. 1h,i) were similar to those of Fig. 1b,c, with the exception that the peak frequency of the reticular lamina was slightly lower than that of the basilar membrane. Thus, in living mouse cochleae, electrically evoked reticular lamina vibration includes a fast broadband component and a delayed sharply tuned component, while the basilar membrane vibration shows only the delayed response.

### Acoustically evoked vibrations in wild-type mice

**Figure 2:**
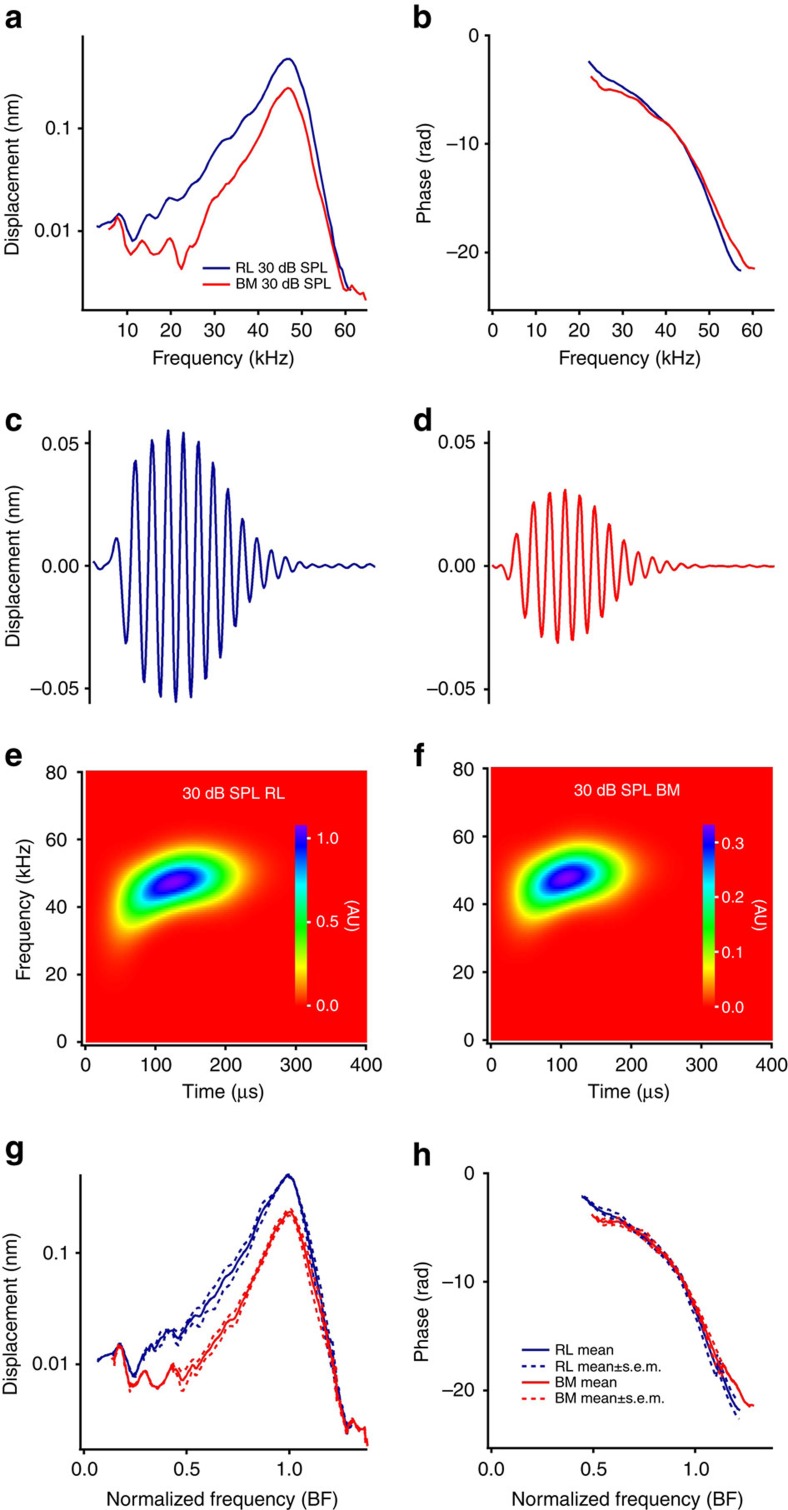
Acoustically evoked reticular lamina and basilar membrane vibrations in wild-type mice. (**a**) Magnitudes of reticular lamina (RL) (blue) and basilar membrane (BM) (red) responses to 30-dB SPL tones as a function of frequency. (**b**) RL and BM phase responses. (**c**) The time waveform of the RL response showed only delayed periodic oscillations without the initial peak. (**d**) Time waveform of the BM is similar to the RL response except for a small magnitude. (**e**,**f**) Spectrograms of the RL and BM only show the delayed tuned component. (**g**,**h**) Grouped magnitudes and phase of the RL (blue) and BM (red). Data are presented as mean±s.e.m. (*n*=7).

To determine the physiological relevance of the reticular lamina and basilar membrane responses to electrical stimulation, we measured acoustically induced responses using 30-dB SPL pure tones (Fig. 2). Contrasting with the responses to electrical stimulation, both reticular lamina and basilar membrane responses to tones were sharply tuned and their phase decreased quickly near the peak frequency (Fig. 2a,b). Time waveforms and spectrograms showed only the delayed tuned component, but no fast broadband response (Fig. 2c–f). Consistent with the electrically evoked responses, grouped acoustically induced reticular lamina vibrations were significantly larger than basilar membrane responses and they were in phase at the BF (Fig. 2g,h). The similarity of acoustically and electrically induced responses indicates that the delayed response caused by electrical stimulation is a conventional cochlear travelling wave.

### Electrically evoked vibrations in *Tecta* ^*C1509G/C1509G*^ mice

**Figure 3:**
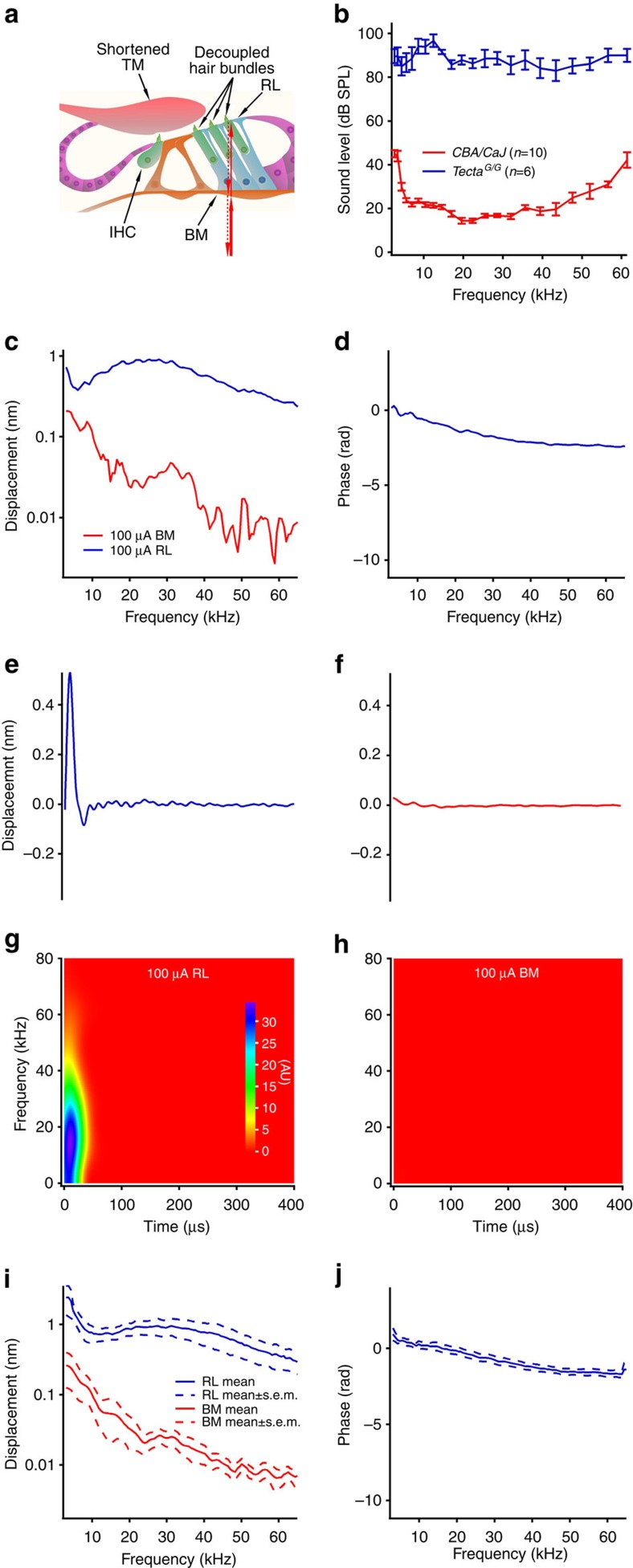
Electrically evoked reticular lamina and basilar membrane vibrations in *Tecta*^*G/G*^ mice. (**a**) Diagram shows the shortened and detached tectorial membrane (TM), which disables mechanoelectrical transduction of outer hair cells and the cochlear feedback system. (**b**) Thresholds of compound action potentials of the auditory nerve. (**c**) Magnitudes of reticular lamina (RL) (blue) and basilar membrane (BM) (red) responses to 100-μA sinusoidal currents as a function of frequency. (**d**) RL phase response. The BM phase is not shown because it was dominated by noise due to the low response amplitude. (**e**) The time waveform of the RL response shows only an initial peak and no delayed periodic oscillation. (**f**) Time waveform of the BM shows no significant response. (**g**) Spectrogram of the RL demonstrates only the fast broadband component. (**h**) Spectrogram of the BM shows no fast or delayed component. (**i**) Grouped magnitudes of the RL (blue) and BM (red) responses to 100-μA currents. (**j**) Grouped RL phase. Data are presented by mean and standard error (mean±s.e.m., *n*=7).

To determine the role of functional cochlear feedback on reverse transduction, we measured electrically evoked reticular lamina and basilar membrane vibrations in *Tecta*^*C1509G/C1509G*^ mice (referred to here as *Tecta*^*G/G*^). Because of deficient mechanoelectrical transduction^38^ and altered tectorial membrane resonance^39^,^40^,^41^,^42^,^43^,^44^ caused by the shortened tectorial membrane (Fig. 3a), these mice have no functional cochlear feedback and suffer from severe hearing loss (blue line in Fig. 3b) but have normal electrically evoked otoacoustic emissions. In these mice, the reticular lamina response to 100-μA current again displayed a large broadband response, but strikingly did not show a response peak or fast phase accumulation near the BF (blue lines in Fig. 3c,d). The basilar membrane vibration (red line in Fig. 3c) was much smaller than the reticular lamina vibration. The time waveform and spectrogram of the reticular lamina response showed only an initial peak and fast broadband response, but no delayed component (Fig. 3e,g); the time waveform and spectrogram of the basilar membrane (Fig. 3f,h) showed no significant response. This observation was confirmed by the grouped data from seven *Tecta*^*G/G*^ mice (Fig. 3i,j). These data unambiguously show that reverse transduction can generate substantial force over all audible frequencies and is not tuned, and demonstrate that the delayed tuned response depends on a functional cochlear feedback system. Furthermore, because the decoupling between hair bundles and the shortened tectorial membrane in *Tecta*^*G/G*^ mice^38^ did not decrease the reticular lamina vibration significantly, the data also suggest that reverse transduction is likely to depend on the somatic motility of the outer hair cells.

### Salicylate reduces electrically evoked vibrations

**Figure 4:**
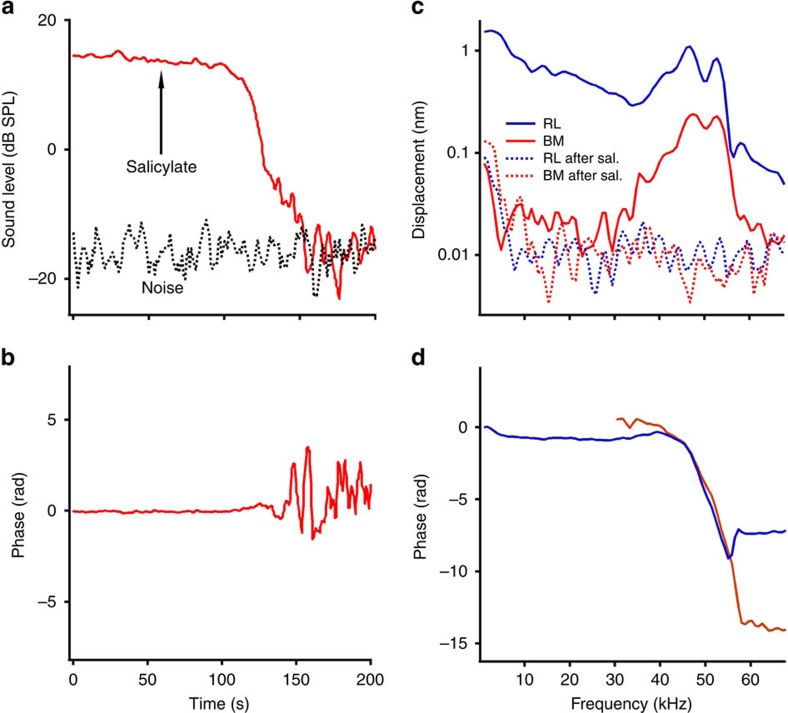
Sodium salicylate suppressed electrically evoked reticular lamina and basilar membrane vibrations. (**a**) Magnitude of acoustically evoked distortion product otoacoustic emission at 27 kHz (red solid line) and the noise floor (black dotted line). Salicylate induced a significant decrease of the otoacoustic emission in less than 3 min. (**b**) The phase of the otoacoustic emission became random when the magnitude approached the noise floor. (**c**) Magnitudes of reticular lamina (RL) and basilar membrane (BM) responses to 100-μA currents before (solid) and after (dotted) sodium salicylate application. Salicylate reduced the RL and BM responses to the noise floor. (**d**) Phase responses of the RL and BM before salicylate application.

We confirmed the role of the somatic motility of outer hair cells by examining the effect of sodium salicylate^45^,^46^. Local application of sodium salicylate reduced acoustically evoked distortion product otoacoustic emissions (Fig. 4a,b), as well as electrically evoked reticular lamina and basilar membrane vibrations (Fig. 4c,d) to the noise floor. Salicylate inhibits outer hair cell somatic motility *in vitro*^47^ and *in situ*^21^, and suppresses electrically evoked otoacoustic emissions and the cochlear amplifier *in vivo*^48^,^49^, likely by interacting with chloride^50^. Our results are thus consistent with somatic motility underlying reverse transduction.

## Discussion

**Figure 5:**
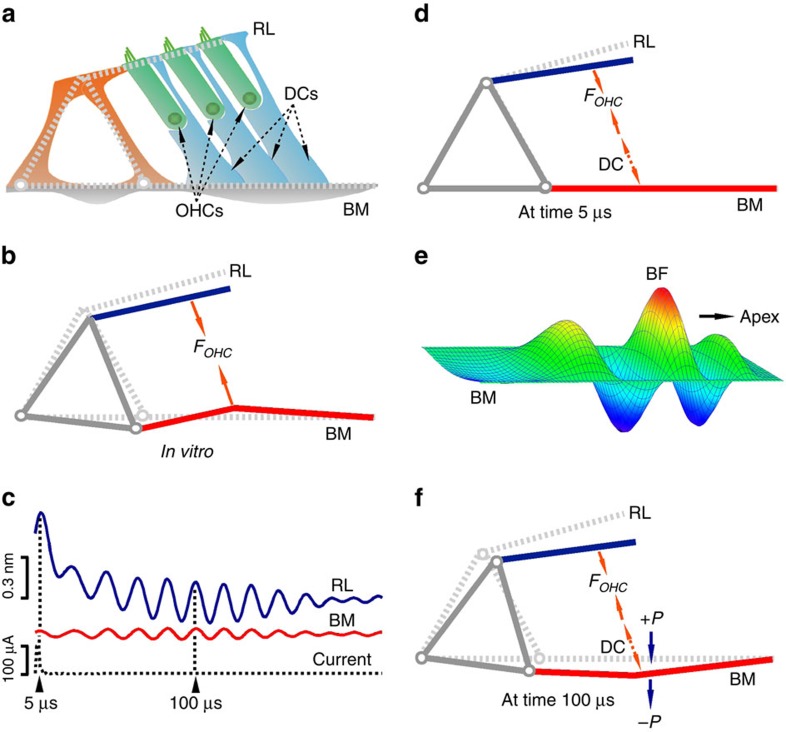
Effects of outer hair cell-generated force on cochlear micromechanics *in vitro* and *in vivo*. (**a**) The structure formed by the triangle of Corti and the reticular lamina (RL) is likely rigid, so outer hair cell (OHC)-generated force can act directly on the RL and indirectly on the basilar membrane (BM) through Deiters' cells (DCs). (**b**) Antiphase motion of the RL and BM recorded *in vitro* indicates that the cellular force pulls the RL and BM together and causes the rigid structure to rotate towards scala tympani^21^. (**c**) Time waveforms of RL and BM responses to a 100-μA electrical impulse in living cochlea. (**d**) An impulse current induced a large fast RL displacement at time 5 μs without significant BM response *in vivo*. (**e**) Like an externally given acoustic click, the electrical impulse evoked a conventional travelling wave, that is, the delayed tuned response. (**f**) At time ∼100 μs, the travelling wave reached the maximum at the best frequency (BF) location. When OHCs rode on the BM, they changed their length in response to membrane voltage variation caused by mechanoelectrical transduction current. The in-phase vibrations of the RL and BM results in a maximum vibration at the apical ends of OHCs. *F*_*OHC*_, OHC force. *+P* and *−P*, the travelling wave-related pressures in scala media (*+P*) and scala tympani (−*P*).

Since the apical surfaces of outer hair cells are a part of the reticular lamina, but their bases connect to the basilar membrane through the Deiters' cells^38^,^51^, force generated by the somatic motility of outer hair cells is coupled directly to the reticular lamina but indirectly to the basilar membrane (Fig. 5a). In their *in vitro* experiments, Mammano and Ashmore^21^ interpreted electrically evoked antiphase motions of the basilar membrane and the reticular lamina in isolated cochlea (Fig. 5b) as direct evidence that cochlear amplification arises from the outer hair cells. Here we studied reverse transduction of outer hair cells in the living cochlea with physiologically relevant stimuli. While membrane potential change of outer hair cells induced by the 33-μA current (∼4 mV; see Methods) is comparable to that caused by ∼19% modulation of the transducer channel conductivity at ∼50 kHz^52^, the actual membrane potential change likely was smaller because the reticular lamina vibration was not saturated at 100-μA current level (Fig. 1b,d), and the directly measured transmembrane potential of outer hair cells induced by a 200-μA current was about 0.5 mV in isolated cochleae^21^. Thus, the large fast broadband response of the reticular lamina *in vivo* (Fig. 1b,d,f,h and the blue line at 5 μs in Fig. 5c) demonstrated that reverse transduction-generated force can drive the reticular lamina over the entire audible frequency range in the living mouse cochlea (Fig. 5d). The negligible fast response of the basilar membrane at frequencies below the BF indicates that the structure of the basal region of the living mouse cochlea is very stiff, that hair cells are coupled inefficiently to the basilar membrane, or both. The delayed sharply tuned response (Figs 1 and 5c at 100 μs) and its similarity to the acoustically evoked response (Fig. 2) indicates that electrical stimulation induces a conventional basilar membrane travelling wave^32^ (Fig. 5e).

The phase relationship between the reticular lamina and basilar membrane vibration is critical because it determines how the cochlea feedback system works^21^,^22^,^40^,^53^,^54^,^55^,^56^,^57^,^58^,^59^,^60^,^61^,^62^. Surprisingly, we found the basilar membrane vibrates in phase with the reticular lamina at the BF. As the basilar membrane vibration travels forward, it reaches its maximum at the BF location. When the outer hair cells ride on the basilar membrane travelling wave, they also change their length in response to the membrane potential change caused by mechanoelectrical transduction current. The in-phase vibration of the reticular lamina and basilar membrane results in maximal vibration at apical ends of outer hair cells (Fig. 5f), which increases hearing sensitivity.

The current *in vivo* data showing that the electrically evoked reticular lamina vibration was larger than the basilar membrane vibration is consistent with previous *in vitro* observations^21^. The in-phase vibrations of the reticular lamina and basilar membrane near the BF observed in the current experiment do not necessarily conflict with observations of antiphase vibration recorded *in vitro* at very low frequencies. Antiphase vibrations could not observed in our experiments because the electrically evoked basilar membrane vibration cannot be detected at very low frequencies due to sharp tuning in living cochleae. While the current magnitude data, which show that the reticular lamina vibration is larger than the basilar membrane vibration, are similar to those in guinea pigs^53^, the present data show no significant phase or BF difference between the reticular lamina and basilar membrane vibrations near the response peak. Compared with the mouse apical micromechanical data^26^,^63^, although vibrations of both the reticular lamina and tectorial membrane were larger than the basilar membrane vibration, the tectorial membrane, but not the reticular lamina vibration, phase leads the basilar membrane phase near the best frequencies. These data suggest a phase difference between the reticular lamina and tectorial membrane, which may correspond to the expected shearing motion as hair bundles are deflected. The inconsistency between the present study and others likely resulted from differences in phase detection techniques, animal species and measurement locations. Despite these differences, all cochlear micromechanical measurements demonstrate a complex mechanical response inside the cochlear partition, which requires further refined measurements to reveal its mechanism and roles in hearing.

In summary, reverse transduction generates substantial force *in vivo* at all audible frequencies, and this remarkably fast cellular force is produced mainly through somatic motility of outer hair cells. Moreover, force elicited by outer hair cells causes a conventional basilar membrane travelling wave rather than simply an immediate local vibration of the basilar membrane. The travelling wave vibrates in phase with the reticular lamina near the BF, which results in maximal vibration at the apical ends of outer hair cells and consequently boosts hearing sensitivity.

## Methods

### Animals

Forty-one CBA/CaJ and 23 *Tecta*^*G/G*^ mice^38^ at age of 3 to 5 weeks of either sex were used in this study. The animal use protocol was approved by the Oregon Health & Science University Institutional Animal Care and Use Committee.

### The custom-built heterodyne low-coherence interferometer

**Figure 6:**
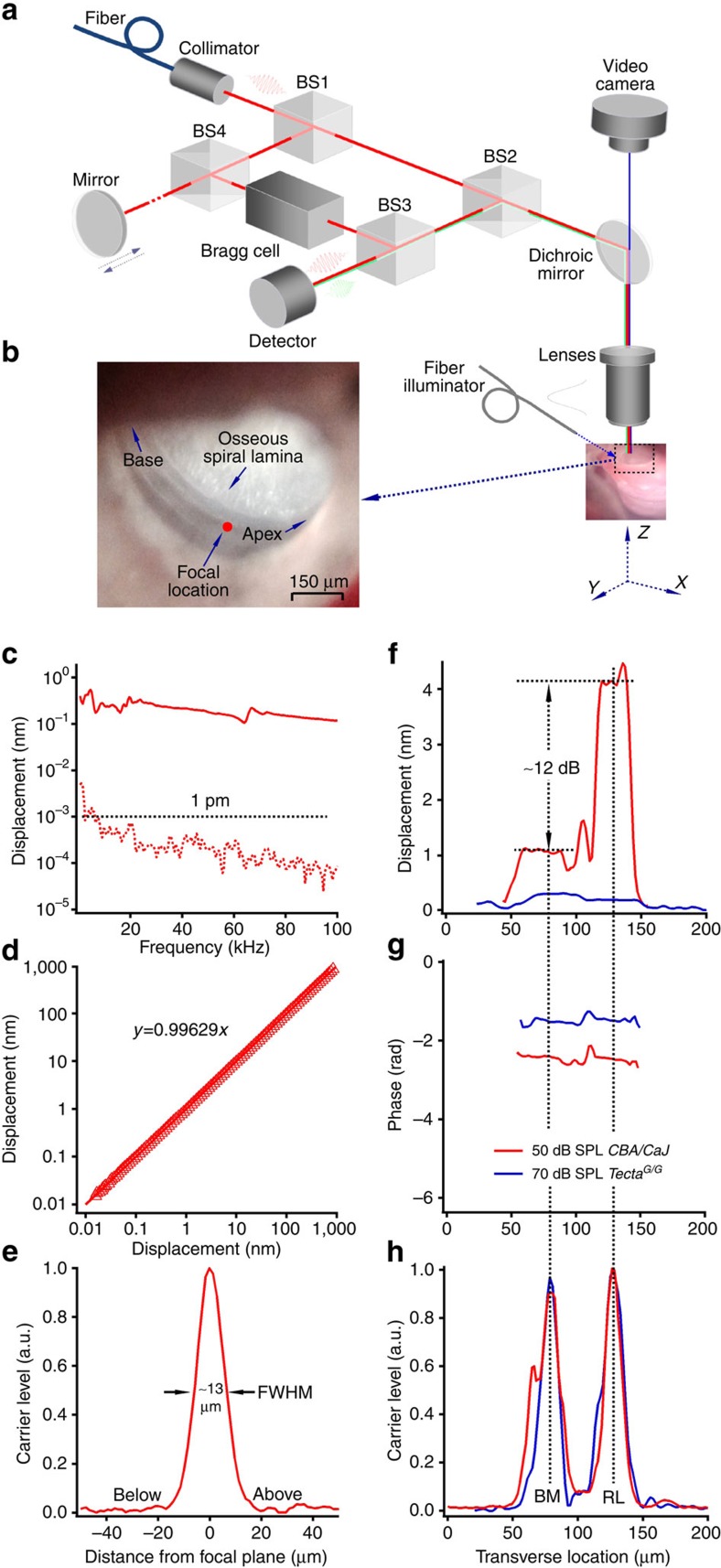
The custom-built heterodyne low-coherence interferometer and its performance. (**a**) Diagram of the interferometer. BS1-4, polarization maintaining beam splitters. (**b**) The intravital image of the cochlear partition taken through the intact round window membrane using the microscope with white light transillumination. The object light beam was focused on the outer hair cell region, which was confirmed visually by changing the focal plane. (**c**) The noise floor of the measurement is <1 pm at frequency above 8 kHz (red dotted line), and the frequency response is up to 100 kHz (red solid line). (**d**) The displacement measured using the low-coherence interferometer increased linearly with vibration magnitude from 0.01 to 1,000 nm. (**e**) The peak of the backscattered light level from a glass surface in air had full-width at half maximum (FWHM) ∼13 μm, equivalent to ∼10 μm in water, which was determined by the coherence length of the light and numerical aperture of the objective lens. (**f**,**g**) Magnitude and phase responses to acoustical stimuli in a wild-type (red) and a *Tecta*^*G/G*^ (blue) mouse as a function of the transverse location. (**h**) The carrier signal peaked at the BM and RL locations.

Different from coherent light, such as laser light, low-coherence light has a constant wavelength and phase only over a small distance along the light beam. Taking advantage of the small coherence length, an interferometer using low-coherence light can measure vibration with a high axial resolution^26^,^53^,^64^,^65^. Low-coherence light with central wavelength of 680 nm and spectral width of ∼10 nm was coupled into a single-mode optic fibre. The collimated light beam was divided into the object and reference beams at polarizing beam splitter BS1 (Fig. 6a). The object light through beam splitter BS2 was focused on the cochlear partition through a long-working-distance objective lens (Plan Apo × 20, numerical aperture (NA) 0.42, Mitutoyo, Japan). Doppler-shifted backscattered light was collected and sent back to beam splitter BS3 through the objective lens and BS2. The reference light with frequency modulated by a Bragg cell was combined with backscattered object light at BS3 and projected onto a photodetector. When the lengths of the object and reference arms were equal, achieved by moving the mirror, interference occurred. The vibration magnitude and direction in the optical axis was derived from the electrical signal of the detector using a digital frequency demodulator.

Sensitivity and frequency response of the interferometer were demonstrated by measuring vibration of a miniature front mirror driven by a piezoelectric actuator through a 2.5-OD neutral density filter. The piezoelectric actuator was calibrated using a laser interferometer. The noise floor was <1 pm at frequencies above 8 kHz (Fig. 6c), and the frequency response was approximately flat up to 100 kHz (solid red curve). The measured displacement increased linearly with vibration magnitude from 0.01 to 1,000 nm (Fig. 6d). The level of the backscattered light, that is, carrier, from a glass surface in air as a function of the distance from the focal location had full-width at half maximum (FWHM) of ∼13 μm, equivalent to ∼10 μm in water (Fig. 6e). FWHM was determined by the coherence length of the light, NA of the objective lens, and refractive index of the media. Thus, our low-coherence interferometer had sufficient sensitivity, frequency bandwidth, and axial resolution for measuring the reticular lamina and basilar membrane vibrations at high frequencies in mouse cochleae.

### Measurement of the cochlear partition vibration

Animal experiments were conducted on a vibration isolation table inside an acoustically attenuated double-wall booth. Under anaesthesia induced by ketamine and xylazine (100 mg kg^−1^ and 10 mg kg^−1^ intraperitoneally), a tracheotomy was performed and natural free breathing was maintained. Body temperature was kept at 38 °C using a heating blanket that was feedback-controlled through a rectal temperature probe. The animal's head was held firmly using a custom-built holder, which was mounted on a three-dimensional translational stage with rotation capability. The surgery for visualizing the cochlear partition through the intact round widow membrane was developed based on previous methods used for gerbils^24^,^66^,^67^ and mice^5^. A ventrolateral surgical approach was used to expose the left bulla and to transect the external ear canal. An acoustic probe connected with two electrostatic speakers (EC1, Tucker-Davis Technologies, Alachua, FL) and a microphone was coupled to the remaining bony ear canal to form a closed sound field. Two tones at f1 and f2 frequencies of 36 and 45 kHz and at 50 dB SPL (referred to 20 μPa) were continuously presented to the ear canal, and the evoked distortion product otoacoustic emission at 2f1–f2 (27 kHz) was displayed on a dynamic signal analyzer (SR785, Stanford Research Systems, Sunnyvale, CA) and recorded through a digital lock-in amplifier (SR830 DSP, Stanford Research Systems, Sunnyvale, CA). No significant change in distortion product otoacoustic emission was assured during experiments. Hearing sensitivity was also confirmed by measuring the compound action potential of the auditory nerve^68^.

When the basilar membrane was positioned approximately in the horizontal plane, a white light beam through an optical fibre was brought close to the lateral bony wall of the scala vestibuli and scala media. With this transillumination, landmarks of the cochlear partition were visible through the intact round window membrane. Under direct visualization, low-coherence light from the interferometer was focused on the center of the outer hair cell region through the long-working-distance objective lens (Fig. 6b). The transverse locations of the basilar membrane and reticular lamina were determined by measuring the backscattered light level as the focal spot scanned along the transverse direction through the outer hair cell region. Scanning was performed by a computer-controlled three-dimensional positioning system^24^,^66^,^67^,^68^. As the focal spot moved from the scala tympani to scala media, the backscattered light level increased and formed a peak (red dotted line on the right in Fig. 1a and red and blue lines in Fig. 6h), where the image of the focal spot of the low-coherence light beam was the sharpest. The light level then decreased and increased, forming the second peak at the reticular lamina location. Because of the transition of refractive index at the interfaces between the cochlear fluid and basilar membrane and between outer hair cells and the tectorial membrane, the backscattered light increased at these locations. The carrier signal was also likely enhanced by the integration of the spatially coherent backscattered light from the tissue in the focal spot, that is, low-coherence-enhanced backscattering^69^. Thus, the two peaks of the carrier signal were used to determine the basilar membrane and reticular lamina locations in the transverse direction. This assignment was supported by the observation that the ∼49-μm separation between the two peaks of carrier signal (Fig. 6h) is comparable to the distance between the basilar membrane and reticular lamina at the base of mouse cochlea (∼50 μm)^38^,^51^. Furthermore, magnitudes and phase of the cochlear partition vibration in response to acoustical stimuli at the basilar membrane location were different from those at the reticular lamina location (Fig. 6f,g), confirming distinct mechanical properties at the two locations.

The current experimental method is advantageous both because the cochlea remained intact, that is, unopened, and because both reticular lamina and basilar membrane vibrations could be measured through the intact round window membrane. This configuration allowed us to study reverse transduction in the healthy cochlea, where cochlear feedback is functional. In addition to its sufficient spatial and temporal resolution, our heterodyne low-coherence interferometer has an exceptionally low noise floor (<1 pm in Fig. 6c). This sensitivity is critical for measuring high-frequency cochlear partition vibration in the mouse cochlea because vibration at the cochlear base is much smaller than that at the apex. Due to its use of the heterodyne principle, our low-coherence interferometer also has a wide dynamic range, no 180-degree phase ambiguity, and high temporal resolution.

### Electrical stimulation of the cochlea

Using a custom-made micro drill, two ∼30-μm holes were made on the lateral bony walls of the scala tympani and scala media at the same longitudinal location as for the vibration measurement at the first turn (∼0.85 mm from the cochlear base). Two electrodes made of 25-μm diameter platinum–iridium wire were used to deliver the electrical current. To avoid high-frequency hearing loss, the electrode in the lateral wall of the scala media was inserted through the bony layer but not soft tissues, while the tip of the other electrode was advanced into the scala tympani's perilymph. Due to the relatively high electrical conductance of the soft tissue and the cochlear fluid, the electrical impedance between the two electrodes was <100 kΩ. Current passing through the electrodes created an electrical field that activated reverse transduction of outer hair cells. To estimate resulting membrane potential change of outer hair cells, current injection-induced AC potential in the scala media was recorded using a micro glass electrode referred to the scala tympani potential^70^ in three CBA/caJ mice. A 33-μA sinusoidal current at 50 kHz, which evoked the cochlear partition vibrations comparable to those induced by ∼30-dB SPL tones, resulted in ∼66-mV scala media AC potential. According to the equivalent electrical circuit for the outer hair cell^52^, assuming that the capacitance of hair-bundle bearing membrane is much smaller than that of the basolateral membranes, the ratio of the resulting membrane AC potential of the outer hair cells (*V*_m_) to the AC potential in the scala media (*V*_sc_) was derived from equation *V*_m_/*V*_sc_=1*/*(1+q*(1+j*f/f_c_)), where *f* is the stimulus frequency, *f*_c_ the cutoff frequency of the outer hair cell, *j* the square root of minus one, and *q* the ratio of the conductance of the hair bundle at rest (*G*_MT0_) to the basolateral conductance (*G*_*K*_). On the basis of established parameters by Johnson *et al*.^52^, the AC membrane potential induced by a 33-μA current was ∼4.1 mV, which is ∼6.2% of the AC potential over the scala media. After the electrodes were fixed on the bony edge of the opened bulla using dental cement, the animal's head was adjusted to the position for vibration measurement.

Although all animals survived anaesthesia and surgery, insensitive cochlear conditions and poor transparency of the round window membrane often prevented us from collecting useful data. Drilling into the cochlea and installing electrodes often caused high-frequency hearing loss. Despite these technical difficulties, sufficient data were collected for this study; micromechanical data reported in this paper were collected from 12 of 41 wild-type mice and 7 of 23 *Tecta*^*G/G*^ mice. Compound action potentials were recorded from 10 wild-type and 6 *Tecta*^*G/G*^ mice.

### Signal generation and data acquisition

A computer-controlled digital lock-in amplifier (SR830 DSP, Stanford Research Systems, Sunnyvale, CA) was used for signal generation and data acquisition. A continuous sinusoidal signal was generated by the internal function generator of the lock-in amplifier. The frequency of the signal was changed from 0.75 to 67.5 kHz by 0.75 kHz per step, and the current level was varied from 100 to 10 μA by ∼10 dB per step. The sinusoidal signal was used to drive an optically isolated current stimulator to generate constant currents. The electrodes were connected to the output of the current stimulator with shielded wire. A 1-kΩ resistor was connected in the output circuit in series, and the current level was monitored by measuring the voltage across the resistor.

Since ∼4-ms electrical pulses were used to stimulate the reverse transduction in the isolated cochlea^21^, we used similar electrical current pulses to stimulate the living cochlea in pilot experiments of this study. Electrical current pulses up to 100 μA did not evoke significant basilar membrane vibration. Currents larger than 100 μA were not used because they distorted the stimulus and produced high-frequency hearing loss. The lack of electrical pulse-induced responses was caused likely by high stiffness and best frequencies of the cochlear partition at the base of the mouse cochlea. At the apex, where the BF and stiffness are low, electrical pulses are expected to produce significant responses.

When the object beam of the interferometer was focused on the basilar membrane, or reticular lamina, electrical or acoustical stimuli at different frequencies and levels were delivered to the cochlea. The magnitude and phase of the output signal at the stimulus frequency from the interferometer were measured by the lock-in amplifier and recorded on a computer.

### Local application of sodium salicylate

After the electrically evoked reticular lamina and basilar membrane vibrations were measured under the normal cochlear condition, a small crystal (∼0.027 mm^3^) of sodium salicylate (Sigma-Aldrich, Inc., Milwaukee, WI) was placed on the round window membrane^48^. Once the effect of sodium salicylate was confirmed by a significant decrease of acoustically evoked distortion product otoacoustic emissions, the electrically evoked reticular lamina and basilar membrane vibrations were measured again. The effect of salicylate on the reverse transduction of the outer hair cells was shown by the difference between the reticular lamina responses before and after the salicylate application.

### Data analysis and statistical methods

Igor Pro (Version 6.35A5, WaveMetrics, Lake Oswego, OR) was used for processing and analysing the data. The frequency bandwidth of the reverse transduction was presented by plotting magnitude and phase of the reticular lamina response as a function of frequency. The delays introduced by the interferometer and the current stimulator were removed from the presented data. To show the temporal response, time waveforms were obtained through inverse fast Fourier transform of the frequency-domain data. The electrical impulse was derived from the magnitude and phase spectra of the current from the stimulator at current level of 100 μA. The electrical impulse is a brief (∼5 μs) pulse (dotted black line in Fig. 1d) not a step input. The spectrograms were produced through Wigner transform of time waveforms. The grouped results were presented by mean and s.e.m. calculated across the animals at given stimulus level, frequency and cochlear conditions.

## Additional information

**How to cite this article:** Ren, T. *et al*. Reverse transduction measured in the living cochlea by low-coherence heterodyne interferometry. *Nat. Commun.* 7:10282 doi: 10.1038/ncomms10282 (2016).
